# Detrimental Effect of Low‐Magnitude High‐Frequency Vibration in an Ex Vivo Model of Intervertebral Disc Degeneration Despite Estrogen Treatment

**DOI:** 10.1002/jsp2.70183

**Published:** 2026-04-22

**Authors:** Jan Ulrich Jansen, Felizitas Figel, Franziska Widmayer, Morten Vogt, Maria Ahrens, Hans‐Joachim Wilke, Anita Ignatius, Melanie Haffner‐Luntzer, Cornelia Neidlinger‐Wilke, Graciosa Quelhas Teixeira

**Affiliations:** ^1^ Institute of Orthopaedic Research and Biomechanics Ulm University Hospital Ulm Germany

**Keywords:** 17β‐oestradiol, inflammation, intervertebral disc degeneration, low‐magnitude high‐frequency vibration, matrix metabolism, organ culture model

## Abstract

**Introduction:**

Postmenopausal females are at increased risk of intervertebral disc (IVD) degeneration due to estrogen decline. While both estrogen supplementation and low‐magnitude high‐frequency vibration (LMHFV) are proposed as therapies for osteoporosis, their effects and potential interactions on IVD degeneration remain unclear. Therefore, the aim of this work was to investigate the individual and combined effects of 17β‐oestradiol (E2) and LMHFV in a papain (PP)‐induced ex vivo model of IVD degeneration.

**Methods:**

Bovine IVDs were degraded with PP (65 U/mL) and subsequently treated with E2 (10^−7^ M), LMHFV (45 Hz, 0.3 g, 20 min/day), or both; untreated discs served as controls. Gene expression was analyzed on Day 8. On Day 21, cell viability, DNA content, extracellular matrix components (glycosaminoglycan [GAG], collagen), inflammatory (interleukin‐6 [IL‐6]) and catabolic (matrix metalloproteinase‐3 [MMP‐3]) markers, and annular stiffness and strength were assessed.

**Results:**

PP digestion reduced cell viability and decreased GAG content, while increasing IL‐6 and MMP‐3 production. Treatment with E2 partially restored *ESR1* expression but did not affect cell viability or matrix metabolism. LMHFV (PP + LMHFV group) significantly increased DNA and GAG content compared with PP alone, whereas the combined treatment (PP + E2 + LMHFV) further elevated IL‐6 and MMP‐3 production. Annular stiffness was higher following PP treatment but was normalized by all subsequent treatments.

**Conclusion:**

In a severely degenerated IVD model, LMHFV promoted catabolic and inflammatory responses, and E2 treatment was insufficient to counteract these effects. These findings suggest that LMHFV, commonly used to support bone health in osteoporotic patients, may pose risks to IVD integrity in advanced degeneration.

## Graphical abstract

Low‐magnitude high‐frequency vibration (LMHFV), with or without 17β‐oestradiol (E2), exacerbated papain‐induced bovine intervertebral disc degeneration, reducing cell viability, impairing matrix production, and increasing inflammation. E2 showed no protective effect against papain‐induced degeneration or LMHFV.

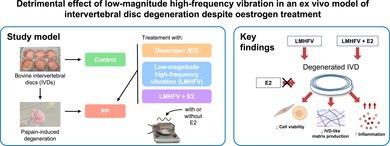

## 1 Introduction

Approximately 80%–85% of the global population experiences LBP during their lifetime [1], with 4%–25% developing chronic disability [2]. Demographic analyses indicate that older women are at slightly higher risk for LBP than age‐matched men [3, 4]. Intervertebral disc (IVD) degeneration (IVDD) is a primary cause of back pain, affecting about 40% of individuals under 30, rising to over 90% in those aged 50–55 [5]. Evidence suggests a link between menopause and lumbar IVDD, implicating estrogen deficiency as a risk factor [4, 6, 7]. Postmenopausal women often show accelerated disc degeneration, likely due to reduced estrogen levels, along with increased incidences of spondylolisthesis and facet joint osteoarthritis [6, 8, 9]. Studies demonstrate that estrogen positively affects IVD metabolism. In vitro, estrogen has been shown to promote IVD cell proliferation [10, 11] and antiapoptotic effects on nucleus pulposus (NP) [12, 13] and endplate [14] cells. In vivo, ovariectomized female rats treated with estrogen displayed healthier IVDs than untreated animals [15]. The mechanism of action of estrogen in the IVD is not fully understood; however, estrogen receptors ERα and ERβ have been detected in human IVD tissues from both male and female DD patients [11, 16, 17], with higher expression of estrogen receptors *ERα* and *ERβ* in degenerated IVDs of males than females [16].

Whole‐body low‐magnitude, high‐frequency vibration (LMHFV) is increasingly being used both as a fitness tool and as a therapeutic intervention for musculoskeletal conditions such as fractures and osteoporosis [18, 19, 20, 21, 22], with studies showing osteoanabolic effects [18, 19, 23]. However, its effects on the IVD might be complex and remain controversial. In mice, short LMHFV exposure (45 Hz, 0.3 g for 30 min) upregulated anabolic genes including aggrecan (*Acan*), collagen type I α1 (*Col1α1*) and *Col2α1*, and downregulated catabolic genes in IVD cells, such as a disintegrin and metalloproteinase with thrombospondin motifs 4 (*Adamts4*), *Adamts5* and matrix metalloproteinase 3 (*Mmp3*). At the protein level, Acan, Col1α1, decorin, and biglycan appeared to be increased [24]. In a 4‐week hindlimb unloading study, daily LMHFV (90 Hz, 0.2 g) preserved disc height and glycosaminoglycan (GAG) content in rats [25]. Additionally, a clinical study found that LMHFV (30 Hz, 0.3 g, 10 min/day) mitigated IVD morphological changes during prolonged bedrest [26]. In contrast, some studies report adverse effects of LMHFV. For example, Jin et al. observed disc and facet joint degeneration following LMHFV (45 Hz, 0.3 g, 30 min/day) in a 10‐week bipedal mouse model [27]. Similarly, Qin et al. reported accelerated cartilage degeneration in an 18‐week rodent study [28]. Widmayer et al. observed a protective effect of E2 against LMHFV‐induced damage in a bovine IVD organ culture model in the absence of a degenerative stimulus [29].

To better understand the effects of E2 and LMHFV on IVDD ex vivo, selecting an appropriate model is essential. Jansen et al. demonstrated that papain (PP) and chondroitinase induce distinct degenerative phenotypes, highlighting the importance of model selection [30]. Although not physiological, PP‐induced models are commonly used to mimic biochemical degeneration through enzymatic degradation of the extracellular matrix, enabling reliable and reproducible induction of degeneration. PP degrades proteoglycans and collagen, leading to impaired biomechanics [30], glycosaminoglycan loss, and reduced hydration, while largely preserving cell viability [31].

Overall, literature suggests that prolonged LMHFV can damage joint tissues [32, 33]; however, estrogen may shift IVD matrix metabolism toward anabolism [34]. Taking this, the main aim of this study was to investigate the combined and individual effects of E2 treatment and LMHFV on IVD metabolism under degenerative conditions.

## 2 Material and Methods

### 2.1 IVD Isolation and Organ Culture Model

**FIGURE 1:**
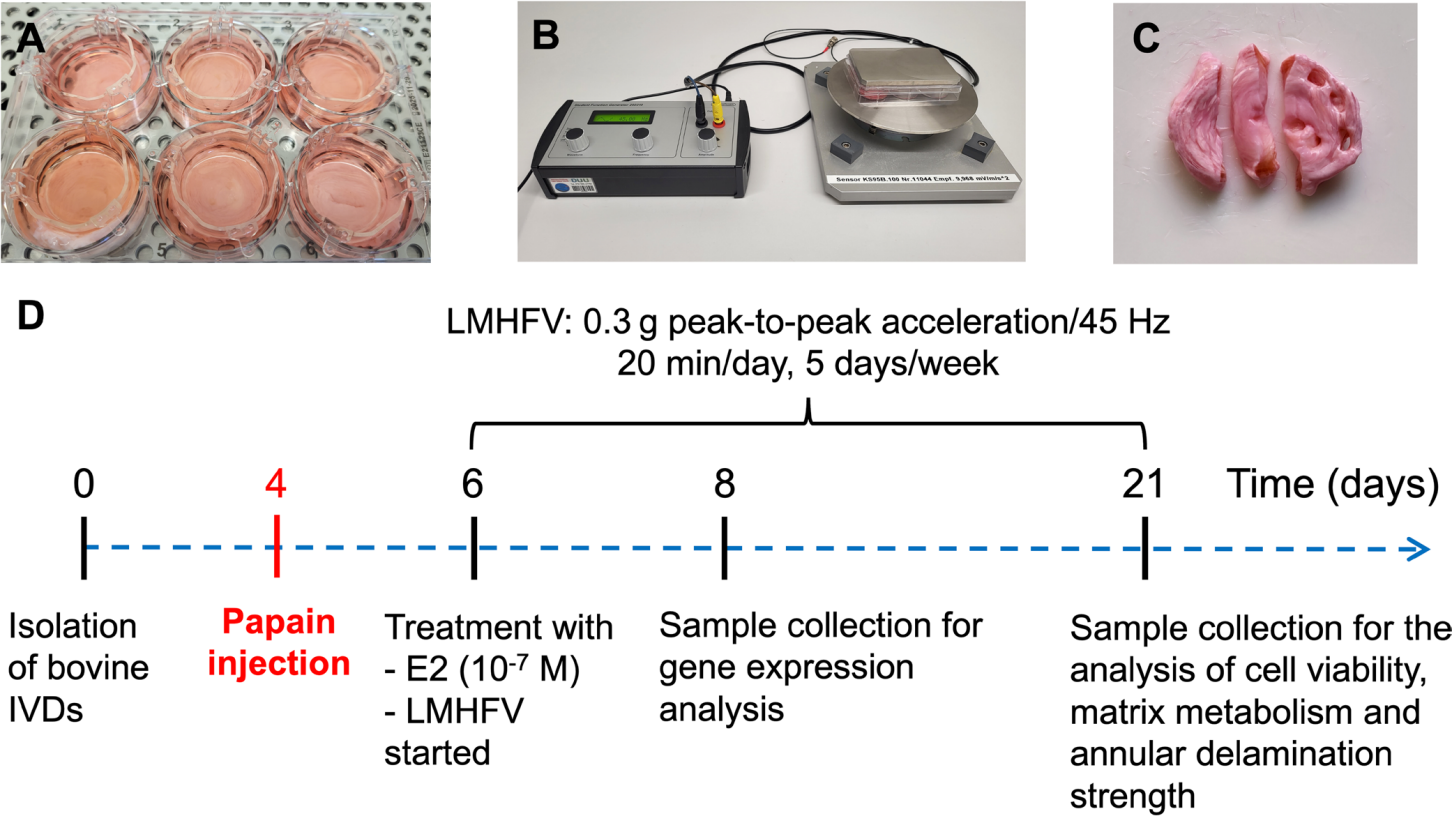
Experimental setup and timeline for ex vivo intervertebral disc (IVD) culture and treatment. (A) Bovine IVDs were isolated and maintained in organ culture using six‐well plates. (B) Low‐magnitude high‐frequency vibration (LMHFV) was applied using a custom‐built vibration platform delivering 0.3 g peak‐to‐peak acceleration at 45 Hz for 20 min per day, 5 days per week. (C) Representative image of a bisected bovine IVD showing the annulus fibrosus and nucleus pulposus regions. (D) Timeline of experimental procedures: IVDs were isolated on Day 0, followed by enzymatic degeneration induced by papain injection on Day 4 (PP group). On Day 6, treatment with 17β‐oestradiol (E2, 10^−7^ M) and/or LMHFV was initiated. Samples were collected on Day 8 for gene expression analysis and on Day 21 for assessment of cell viability (resazurin assay, live/dead staining), biochemical composition (DNA, GAG, and collagen content), histology, and biomechanical properties (peeling test).

Tails from male cattle (12–24 months) were obtained from a local slaughterhouse (*Fleischmarkt Donautal*, Ulm, Germany) and processed as described previously [35]; ethical approval was not required. The experimental design is shown in Figure 1. Briefly, muscles and ligaments were removed, and six IVDs per tail were isolated. IVDs were cultured as previously reported [35]. A membrane filter insert was placed on each IVD, and a static load of 0.46 MPa was applied to prevent swelling during culture (37°C, 6% O_2_, 8.5% CO_2_, saturated humidity). The medium was changed on Day 1 and every second day thereafter. On Day 4, PP (13 U/IVD, 200 μL) was injected into the NP to induce degeneration (PP group) [36]. From Day 6, IVDs (*n* = 6–12) were treated with 100 nM 17β‐E2 (PP + E2), LMHFV (PP + LMHFV), or both (PP + E2 + LMHFV). E2 (100 nM) was added at each medium change [29, 37]. LMHFV (45 Hz, 0.3 g) was applied 20 min/day, 5 days/week using a custom‐built vibration platform [29, 38]. Untreated IVDs served as controls. AF and NP samples were collected after 2 or 14 days of treatment, corresponding to culture Days 8 or 21. Gene expression was assessed on Day 8, while mitochondrial activity was measured on Days 8 and 21. Cell viability, matrix metabolism, and annular delamination strength were investigated on Day 21.

### 2.2 Pressure Measurements in the NP During LMHFV

Intradiscal pressure (IDP) was measured using flexible fiber‐optic pressure sensors (FOP‐LS‐2FR‐30, FISO Technologies, Québec, Canada; 0.6 mm diameter) inserted into the NP of isolated bovine IVDs. Sensor placement was confirmed by X‐ray (Faxitron 43855A, Hewlett Packard, Palo Alto, CA, USA). To prevent displacement during measurement, sensors were secured using a custom‐made clamping device fixed to the six‐well plate. IDP was recorded at 125 Hz under static loading and during LMHFV application. Data from a 10‐s window were processed using a custom MATLAB script (R2017b, MathWorks, USA). Mean pressure and root‐mean‐square amplitude were calculated, and amplitude spectra were derived via Fast Fourier Transform.

### 2.3 Gene Expression Analysis

AF and NP tissues were collected for RNA isolation and gene expression analysis. Total RNA was extracted by homogenizing tissues in 1 mL QIAzol lysis reagent (Qiagen, Düsseldorf, Germany), followed by chloroform phase separation (200 μL) and centrifugation at 12 000 *g* for 30 min at 4°C. The aqueous phase was processed using the PureLink RNA Mini Kit with on‐column DNase treatment (Thermo Fisher Scientific, Waltham, MA, USA). RNA was eluted in 30 μL RNase‐free water, and concentration was measured spectrophotometrically (Spark, Tecan, Männedorf, Switzerland). cDNA was synthesized using the Omniscript RT Kit (Qiagen). Gene expression was quantified by qPCR [29] using either Platinum SYBR Green qPCR SuperMix‐UDG (Invitrogen) with custom primers (Biomers, Ulm, Germany; Table 1) or TaqMan Gene Expression Assays with Fast Advanced Master Mix (Applied Biosystems, Waltham, MA, USA). Reactions were run in duplicate, and melt‐curve analysis confirmed specificity. Data were normalized to glycerinaldehyd‐3‐phosphat‐dehydrogenase (*GAPDH*) and expressed as fold‐change using the 2^−ΔΔCt^ method [39].

**TABLE 1:** Bovine oligonucleotide primers used for qPCR. Custom‐designed primers are indicated by their sequences, while those with an assay ID number were obtained from *Applied Biosystems*.

Gene	Sequence (forward and reverse primer)	Product size (bp)
ACAN	fw: 5′‐ACA GCG CCT ACC AAG ACA AG‐3′ rev: 5′‐ACG ATG CCT TTT ACC ACG AC‐3′	155
*COL1A1*	fw: 5′‐TGA GAG AGG GGT TGT TGG AC‐3′ rev: 5′‐AGG TTC ACC CTT CAC ACC TG‐3′	142
*COL2A1*	5′‐CCT GTA GGA CCT TTG GGT CA‐3′ 5′‐ATA GCG CCG TTG TGT AGG AC‐3′	145
*ESR1*	fw: 5′‐GCC TCA AAT CCA TCA TCT TGC T‐3′ rev: 5′‐CGG TGG ATG TGG TCC TTC TC‐3′	100
*ESR2*	fw: 5′‐CTC CTG GAC ACC TCT CTC CTT TAG‐3′ rev: 5′‐GGT TTC ACG CCA AGG ACT CTT‐3′	85
*GAPDH*	fw: 5′‐ACC CAG AAG ACT GTG GAT GG‐3′ rev: 5′‐CAA CAG ACA CGT TGG GAG TG‐3′	178
*MMP1*	fw: 5′‐ATG CTG TTT TCC AGA AAG GTG G‐3′ rev: 5′‐TCA GGA AAC ACC TTC CAC AGA C‐3′	193
*MMP3*	fw: 5′‐CTG CGG ATA CTT CCA CAG GT‐3′ rev: 5′‐ATG GAT GAG CAG GGA AAC AC‐3′	198
*PTGS2*	Bt03214492_m1	
*TIMP1*	fw: 5′‐GCT GGA CAT TGG AGG AAA GA‐3′ rev: 5′‐CGT CCG GAG AGG AGA TGT AG‐3′	209
*TIMP2*	fw: 5′‐TGA GAG AGG GGT TGT TGG AC‐3′ rev: 5′‐AGG TTC ACC CTT CAC ACC TG‐3′	142
*TP53*	fw: 5′‐ATT TAC GCG CGG AGT ATT TG‐3′ rev: 5′‐CCA GTG TGA TGA TGG TGA GG‐3′	174

Abbreviations: fw, forward; rev, reverse.

### 2.4 Cell Viability

Cell viability was assessed using a LIVE/DEAD cell imaging kit (Invitrogen) and confocal microscopy [35]. AF and NP tissues were rinsed in PBS and phenol red–free DMEM (Gibco), then stained with live (green) and dead (red, BOBO‐3‐iodide) cell indicators for 45 min at 37°C in the dark. After washing, tissues were imaged within 2 h using a confocal microscope (TCS SP8, Leica Microsystems, Wetzlar, Germany). Live cells fluoresced green (488/515 nm), and dead cells red (570/602 nm). Images were analyzed with ImageJ (version 1.53), and viability was expressed as the percentage of live cells.

### 2.5 DNA, GAG, and Collagen Quantification

AF and NP tissues were digested overnight at 56°C with 0.5 mg/mL proteinase K solution (Sigma‐Aldrich) [29, 40]. DNA content in digested tissue samples was quantified using the Quant‐iT PicoGreen dsDNA Kit (Invitrogen) according to the manufacturer's instructions and previously described methods [29, 40]. GAG was quantified in the tissue digests and culture supernatants using the dimethylmethylene blue assay, as previously described [29, 40]. Chondroitin sulfate (Sigma‐Aldrich) was used to generate a standard curve. Absorbance was measured at 525 nm (Spark, Tecan). Total collagen content was estimated via hydroxyproline quantification [29]. For this, 100 μL of tissue digest was incubated with an equal volume of 37% hydrochloric acid (Sigma‐Aldrich) at 110°C for 24 h [29, 41]. Following centrifugation at 10 000*g* for 3 min, 2 μL of the supernatant was transferred to a 96‐well plate and evaporated at 60°C. Hydroxyproline content was determined following the manufacturer's instructions (Sigma‐Aldrich). Considering hydroxyproline constitutes approximately 10% of total collagen mass [42, 43], collagen content was estimated accordingly.

### 2.6 Histology

For histopathological analysis, disc samples were fixed in formalin for 48 h and embedded in paraffin. Sagittal sections (6 μm) were stained with safranin‐O/fast green to assess proteoglycan content [29]. Sections were incubated at 60°C for 15 min, deparaffinized in xylene, rehydrated through graded ethanol, and rinsed in distilled water. Staining was performed sequentially with Weigert's iron hematoxylin (Waldeck, Münster, Germany), 0.01% fast green (Waldeck), and 0.1% safranin‐O (Sigma‐Aldrich), with brief differentiation in 1% hydrochloric acid and 1% acetic acid (VWR, Radnor, PA, USA) between steps. Sections were dehydrated through graded ethanol and xylene (Thermo Fisher Scientific) and mounted with a nonaqueous medium (Vitro‐Clud, R. Langenbrinck, Emmendingen, Germany). Images were acquired by bright‐field microscopy (Axiophot, Zeiss, Jena, Germany).

Collagen distribution was assessed using a picrosirius red staining kit (Abcam, Cambridge, UK). Sections were stained for 1 h at room temperature and visualized under polarized light microscopy (Axiophot, Zeiss), where thin fibers appeared green and thicker fibers appeared yellow to red.

### 2.7 Immunohistochemistry

Immunostaining for IL‐6 and MMP‐3 was performed as previously described [29, 40, 44]. Antigen retrieval was carried out in 10 mM citrate buffer (pH 6.0) at 95°C for 20 min, followed by cooling. Endogenous peroxidase activity was quenched with 3% H_2_O_2_ in tris‐buffered saline (TBS) for 20 min. Sections were blocked in TBS containing 0.1% Tween‐20 (Thermo Fisher Scientific) and 5% goat serum (Jackson ImmunoResearch, West Grove, PA, USA) for 1 h, then incubated overnight at 4°C with rabbit anti‐IL‐6 (Bioss, Woburn, MA, USA; 5 μg/mL) or rabbit anti‐MMP‐3 (Abcam, Cambridge, UK; 5 μg/mL). Negative controls received rabbit polyclonal IgG (Jackson ImmunoResearch; 5 μg/mL). After washing, sections were incubated with biotinylated goat antirabbit IgG (Invitrogen; 5 μg/mL) for 30 min, and signals were visualized using the VECTASTAIN Elite ABC‐HRP and Vector NovaRED kits (Vector Laboratories, Newark, CA, USA). Nuclei were counterstained with hematoxylin, dehydrated through graded ethanol and xylene, and mounted in a nonaqueous medium. AF and NP regions were imaged under bright‐field microscopy. ImageJ analysis was performed as described previously [45], with manual region‐of‐interest selection to exclude artifacts. Images were deconvolved into hematoxylin and DAB channels using the H‐DAB plugin, and staining intensity was quantified as the percentage of DAB‐positive area.

### 2.8 AF Peel Test

The interlaminar strength of the AF tissue was evaluated in all groups using a peel test, as described in previous studies [44, 46]. Annular rings from the IVDs were collected on Day 21 and stored at −20°C. For testing, rectangular AF segments of known width were dissected into a ‘Y’ configuration along a central lamella boundary and clamped into a ‘T’ configuration in a uniaxial material testing machine (Z01, Zwick Roell, Ulm, Germany). The clamped tips were pulled apart along the lamellae at a constant speed of 0.5 mm/s until complete separation of the specimen. A customized program (Matlab 2022, MathWorks Inc., Natick, MA, USA) was used to standardize the analysis by calculating the force profile normalized to the specimen width (N/mm). The program automatically determined the increasing slope phase and the subsequent plateau phase, which are characteristic of peel tests.

### 2.9 Statistical Analyses

Sample size was determined using power analysis with G*Power software (version 3.1.9.6) for a two‐tailed *t*‐test (*α* = 0.05, power = 0.8). Based on preliminary data [29], an expected effect size d of 2.0 indicated a minimum of six samples per group for gene expression analysis, while an effect size of 1.6 required eight samples per group for protein quantifications. Statistical analyses were performed using GraphPad Prism version 10.2.3 (GraphPad Software, La Jolla, CA, USA). For normally distributed data, Brown–Forsythe and Welch one‐way ANOVA followed by Dunnett's multiple comparisons test was used. Nonparametric data were analyzed using the Kruskal–Wallis test with Dunn's multiple comparisons. Pressure measurements were evaluated using the Wilcoxon signed‐rank test. A *p* < 0.05 was considered statistically significant.

## 3 Results

### 3.1 Pressure Measurements in the NP During LMHFV

**FIGURE 2:**
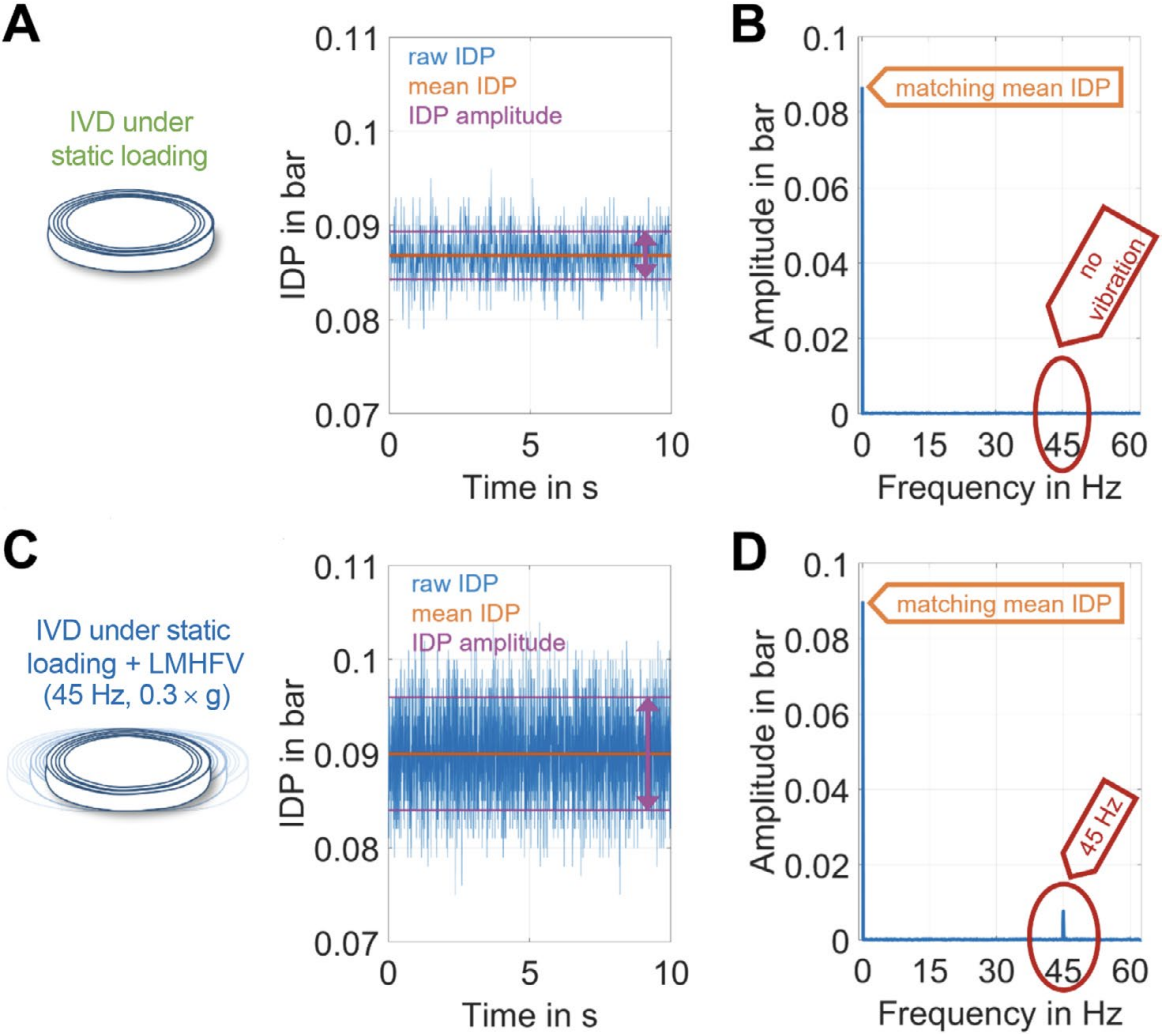
Representative comparison of intradiscal pressure (IDP) in intervertebral discs (IVDs) cultured under static loading alone or under low‐magnitude high‐frequency vibration (LMHFV). (A) Representative IDP results from a control IVD under static loading, showing raw IDP in blue, mean IDP in orange, and mean amplitude denoted by a pink arrow. (B) Corresponding amplitude spectrum of the data shown in (A). The magnitude of the first peak matched exactly with the mean IDP of (A), and no other peaks were observed. (C) Representative IDP results over time from an IVD treated with LMHFV, showing raw IDP in blue, mean IDP in orange, and mean amplitude indicated by a pink arrow. (D) Corresponding amplitude spectrum of the data shown in (C). The magnitude of the first peak matched the mean IDP from (C), and an additional peak was observed at 45 Hz, corresponding to the LMHFV stimulation frequency.

The intradiscal pressure (IDP) of fresh IVDs was not affected by LMHFV (0.087 bar vs. 0.09 bar, *p* > 0.05), but LMHFV significantly increased the mean amplitude (0.0056–0.011 bar, *p* = 0.016). In fresh discs without vibration, the amplitude spectrum showed a single peak at 0 Hz, corresponding to the constant IDP signal with fluctuations representing random noise. With LMHFV, an additional peak appeared at 45 Hz, corresponding to the vibration frequency, while the 0 Hz peak remained unchanged. After 21 days of culture, results were similar: LMHFV did not alter the mean IDP (0.11 bar vs. 0.10 bar, *p* > 0.05) but produced oscillations at 45 Hz in the amplitude spectrum, with no other frequency peaks detected. These findings indicate that LMHFV induces IDP oscillations at the applied frequency without changing the mean IDP, and the effect was reproducible in both fresh and cultured IVDs (Figure 2).

### 3.2 Effect of E2 and LMHFV on Gene Expression

**FIGURE 3:**
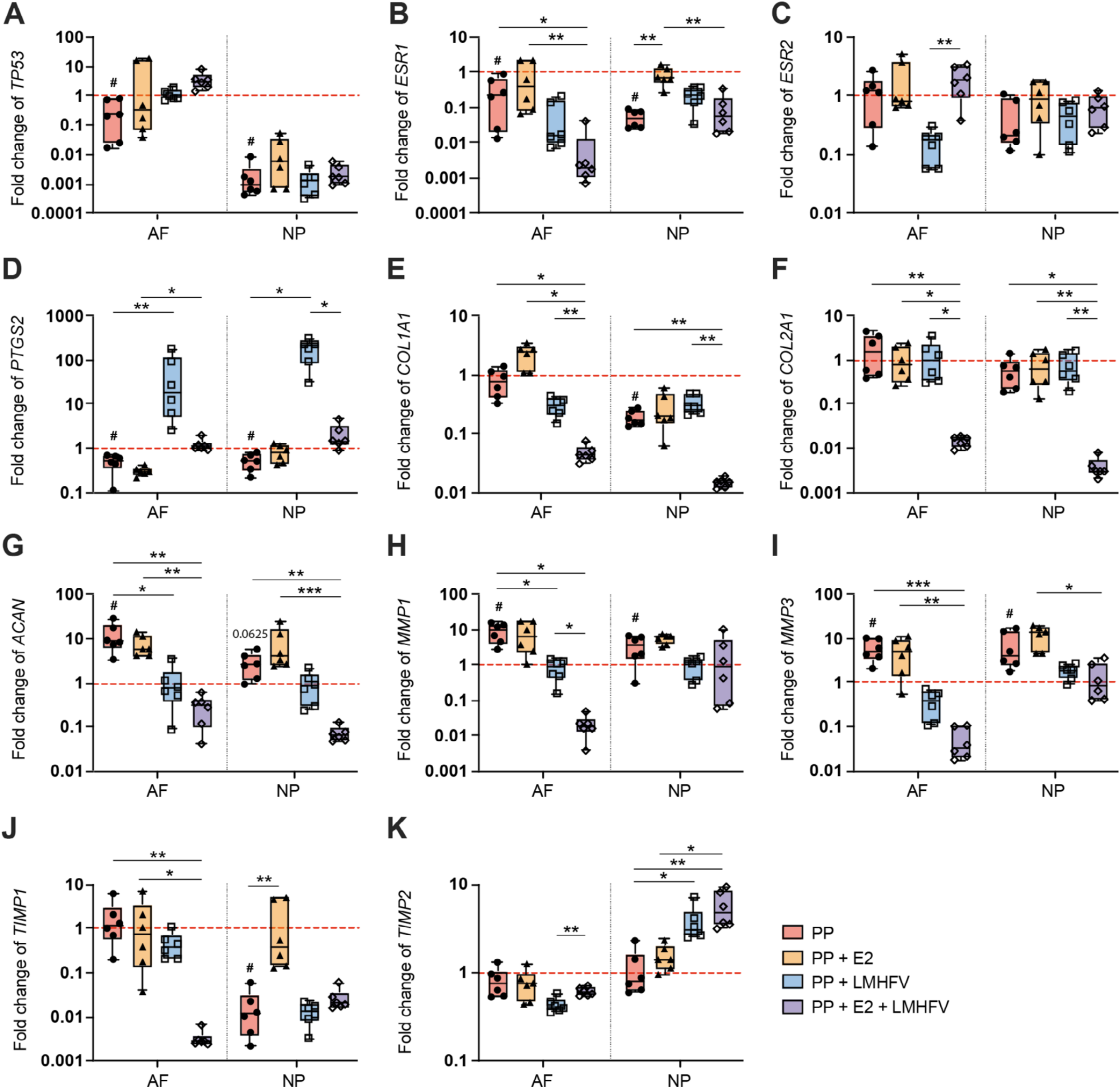
Relative gene expression levels for various targets in the cells of the annulus fibrosus (AF) and nucleus pulposus (NP) at Day 8 of organ culture. (A) Apoptosis regulator *TP53*, (B) estrogen receptor *ESR1*, (C) *ESR2*, (D) mechano‐sensitive prostaglandin‐endoperoxide synthase 2 (*PTGS2*), matrix components (E) collagen type I (*COL1A1*), (F) collagen type II (*COL2A1*), and (G) aggrecan (*ACAN*), matrix metalloproteinases (H) *MMP1*, and (I) *MMP3*, and tissue inhibitors of metalloproteinases (J) *TIMP1* and (K) *TIMP2*. IVD groups were cultured under basal conditions (control), treated with papain (PP) to induce enzymatic digestion, or further treated with estrogen (PP + E2), low‐magnitude high‐frequency vibration (PP + LMHFV), or a combination of both (PP + E2 + LMHFV). mRNA levels were normalized to GAPDH and are expressed relative to the control group (indicated by the red dashed line, set at 1). The results are presented using the 2^−ΔΔCt^ method. Data are presented as box plots showing the median ± interquartile range (*n* = 6 donors per group). ^#^ *p* < 0.05 (^#^Comparison to control); *p < 0.05, **p < 0.01, ***p < 0.001 (*Comparison between treatment groups).

Gene expression related to cell survival, hormone signaling, inflammation, and matrix remodeling in AF and NP was assessed after 2 days of treatment of PP‐digested bovine IVDs with E2, LMHFV, or their combination (E2 + LMHFV). Distinct, tissue‐ and gene‐specific regulatory patterns were observed in response to the treatments. The apoptosis regulator *TP53* (Figure 3A) was significantly downregulated in both AF and NP following PP digestion compared to controls (*p* < 0.05), and this effect was not altered by any of the treatments. Estrogen receptor *ESR1* (Figure 3B) was significantly downregulated in both AF and NP following PP digestion and further suppressed by the combination of PP + E2 + LMHFV (*p* < 0.05). In contrast, E2 alone restored *ESR1* expression in both AF and NP to near control levels. *ESR2* (Figure 3C), on the other hand, showed relatively stable expression across most groups, with a notably lower expression in LMHFV‐treated samples compared to PP + E2 + LMHFV in the AF (*p* < 0.01). *PTGS2*, a gene associated with inflammation and mechanotransduction, was significantly downregulated by PP in both AF and NP cells (*p* < 0.05; Figure 3D). LMHFV treatment increased *PTGS2* expression (*p* < 0.05), whereas the combination of PP + E2 + LMHFV attenuated the LMHFV‐induced upregulation, particularly in the NP (*p* < 0.05).

*COL1A1* expression was unchanged in the AF after PP digestion but significantly downregulated in the NP (*p* < 0.05; Figure 3E). Notably, the combined treatment (PP + E2 + LMHFV) further reduced *COL1A1* expression in both the AF and NP compared to PP alone and to PP + LMHFV groups (*p* < 0.05). *COL2A1* was significantly downregulated in the PP + E2 + LMHFV group compared to PP and the single‐treatment groups (*p* < 0.05; Figure 3F). PP significantly upregulated the expression of *ACAN* in the AF (*p* < 0.05; Figure 3G), with the NP following a similar trend (*p* = 0.0625), whereas E2 + LMHFV treatment of PP‐digested samples downregulated it (*p* < 0.01). *ACAN* was also significantly downregulated in the AF in the PP + LMHFV group (*p* < 0.05).

The matrix‐degrading enzymes *MMP1* (Figure 3H) and *MMP3* (Figure 3I) were upregulated in both AF and NP following PP digestion (*p* < 0.05). In the AF, expression of *MMP1* and *MMP3* was significantly reduced after treatment with PP + E2 + LMHFV compared to PP alone (*p* < 0.05). Expression of tissue inhibitors of MMPs, *TIMP1* (Figure 3J) and *TIMP2* (Figure 3K), showed mixed regulation. In the AF, *TIMP1* was unchanged after PP digestion but was significantly downregulated following PP + E2 + LMHFV compared to both PP alone (*p* < 0.01) and PP + E2 (*p* < 0.05). In the NP, TIMP1 levels were significantly lower in the PP group than in the controls (*p* < 0.05), whereas treatment with PP + E2 restored *TIMP1* expression to control levels (*p* < 0.01). *TIMP2* expression was not affected by PP digestion in either AF or NP but was significantly upregulated in the NP by LMHFV alone or in combination with E2 compared to PP (*p* < 0.05).

### 3.3 Cell Viability and DNA Content

**FIGURE 4:**
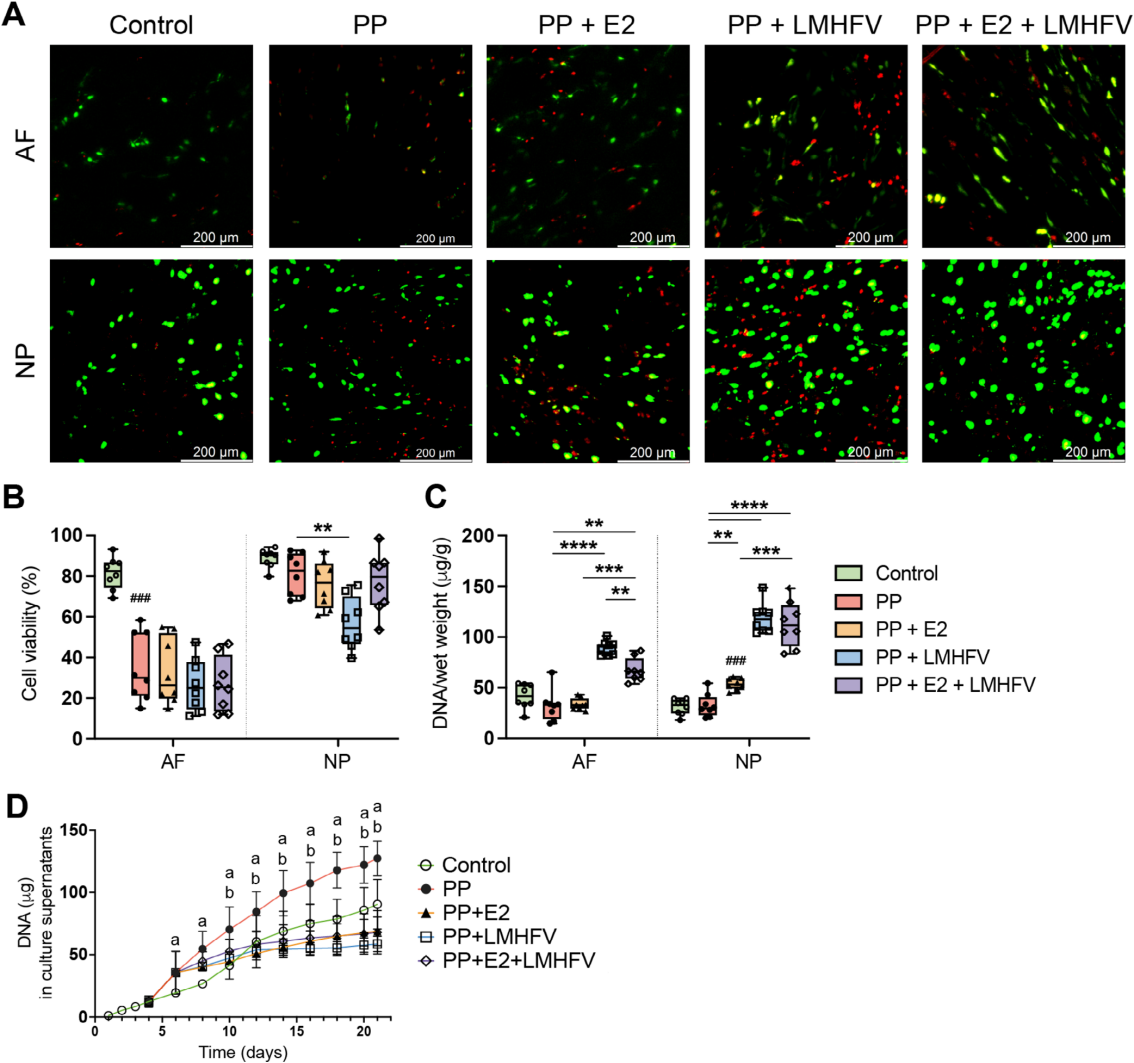
Cell viability in the annulus fibrosus (AF) and nucleus pulposus (NP) across different treatment groups: Control, papain‐treated (PP), PP with estrogen (PP + E2), PP with low‐magnitude high‐frequency vibration (PP + LMHFV), and the combination of PP + E2 + LMHFV. (A) Representative confocal microscopy images of live/dead staining depicting cell viability, with dead cells stained red, and living cells green (scale bars, 200 μm). (B) Cell viability (%) at Day 21 of culture. (C) DNA content in the AF and NP tissues normalized to wet weight (μg/g) at Day 21 of culture. (D) Amount of DNA (μg) released into the culture supernatants. Results are presented in box plots or *xy* plots (*n* = 8 donors per group). ^#^ *p* < 0.05 (^#^Comparison to control); *p < 0.05, **p < 0.01, ***p < 0.001 (*Comparison between treatment groups); ^a^ *p* < 0.05 (^a^Comparison to control); ^b^ *p* < 0.05 (^b^Comparison to PP).

Cell viability was assessed on Day 21, with representative live/dead staining images showing live cells in green and dead cells in red (Figure 4A; separated channels in Figure S1). PP digestion significantly reduced cell viability in the AF (*p* < 0.001; Figure 4B). None of the co‐treatments improved viability in the AF, whereas in the NP, LMHFV further decreased cell viability compared to PP alone (*p* < 0.01).

DNA content seemed to be unaffected by PP digestion in both AF and NP tissues (Figure 4C). In the AF, LMHFV and the combination of E2 + LMHFV significantly increased DNA content relative to PP (*p* < 0.0001 and *p* < 0.01, respectively). In the NP, all treated groups showed higher DNA content than PP alone (*p* < 0.01). Analysis of DNA released into culture supernatants over 21 days revealed the greatest loss in the PP‐treated group compared to controls (*p* < 0.05; Days 6–21) and all treatment groups (*p* < 0.05; Days 10–21; Figure 4D).

### 3.4 GAG and Collagen Content

**FIGURE 5:**
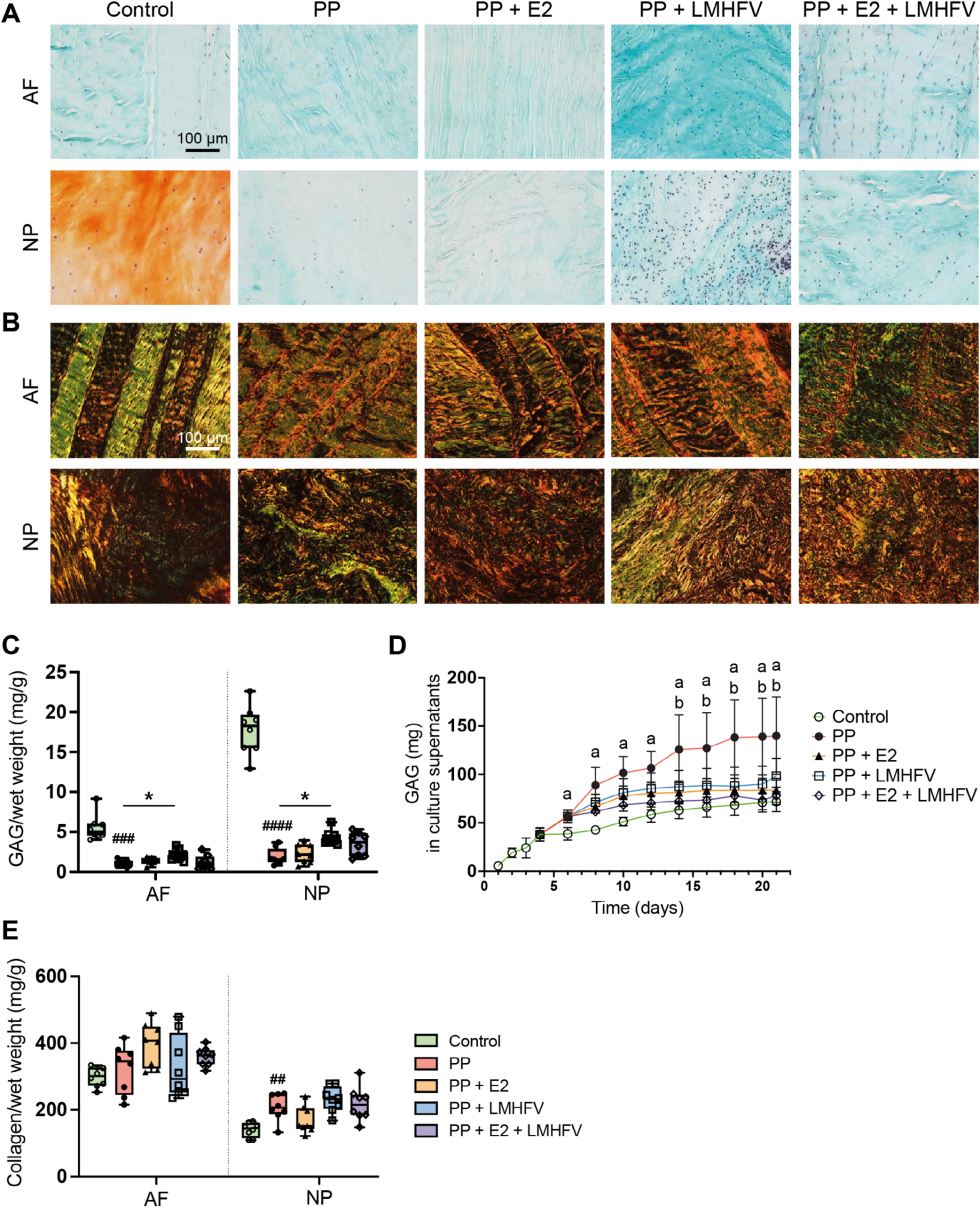
Protein quantification in the annulus fibrosus (AF) and nucleus pulposus (NP) across different treatment groups: Control, papain‐degraded (PP), PP with estrogen (PP + E2), PP with low‐magnitude high‐frequency vibration (PP + LMHFV), and the combination of PP + E2 + LMHFV. Sagittal IVD sections stained with (A) safranin O/fast green for the detection of proteoglycans (proteoglycans are stained orange and collagens are counterstained green; scale bar, 100 μm), and with (B) picrosirius red staining for the detection of collagens. The color range visualized under polarized light microscopy corresponds to relative fiber thickness from thin and less mature green fibers to increasingly thicker and more mature yellow, orange and red fibers (scale bar, 200 μm). (C) GAG content in the AF and NP normalized to wet weight (mg/g). (D) GAG (mg) released into culture supernatants. (E) Collagen content in the AF and NP normalized to wet weight (mg/g). Data are presented in box plots or *xy* plots (*n* = 8 donors per group). ^#^ *p* < 0.05, ^##^ *p* < 0.01, ^###^ *p* < 0.001, ^####^ *p* < 0.0001 (^#^Comparison to control); ^a^ *p* < 0.05 (^a^Comparison to control), ^b^ *p* < 0.05 (^b^Comparison to PP).

Proteoglycan and collagen distribution were first assessed qualitatively using safranin‐O/fast green and picrosirius red staining (Figure 5A,B). In controls, the NP showed strong orange safranin‐O staining, indicating the presence of proteoglycans, which were largely lost after PP digestion (Figure 5A). These observations were supported by biochemical quantification of GAG content on Day 21 (Figure 5C), showing significant reductions in both AF (*p* < 0.001) and NP (p < 0.0001) compared to controls. PP + LMHFV treatment partially restored GAG levels in the NP compared to PP alone (*p* < 0.05). Cumulative GAG release into the culture medium over 21 days was highest in the PP group compared to controls (*p* < 0.05; Days 6–21) and all treatment groups (*p* < 0.05; Days 14–21; Figure 5D).

Total collagen content on Day 21 (Figure 5E) remained unchanged in the AF across all groups, whereas NP collagen levels were significantly increased following PP digestion (*p* < 0.05).

### 3.5 Inflammation and Matrix Degradation

**FIGURE 6:**
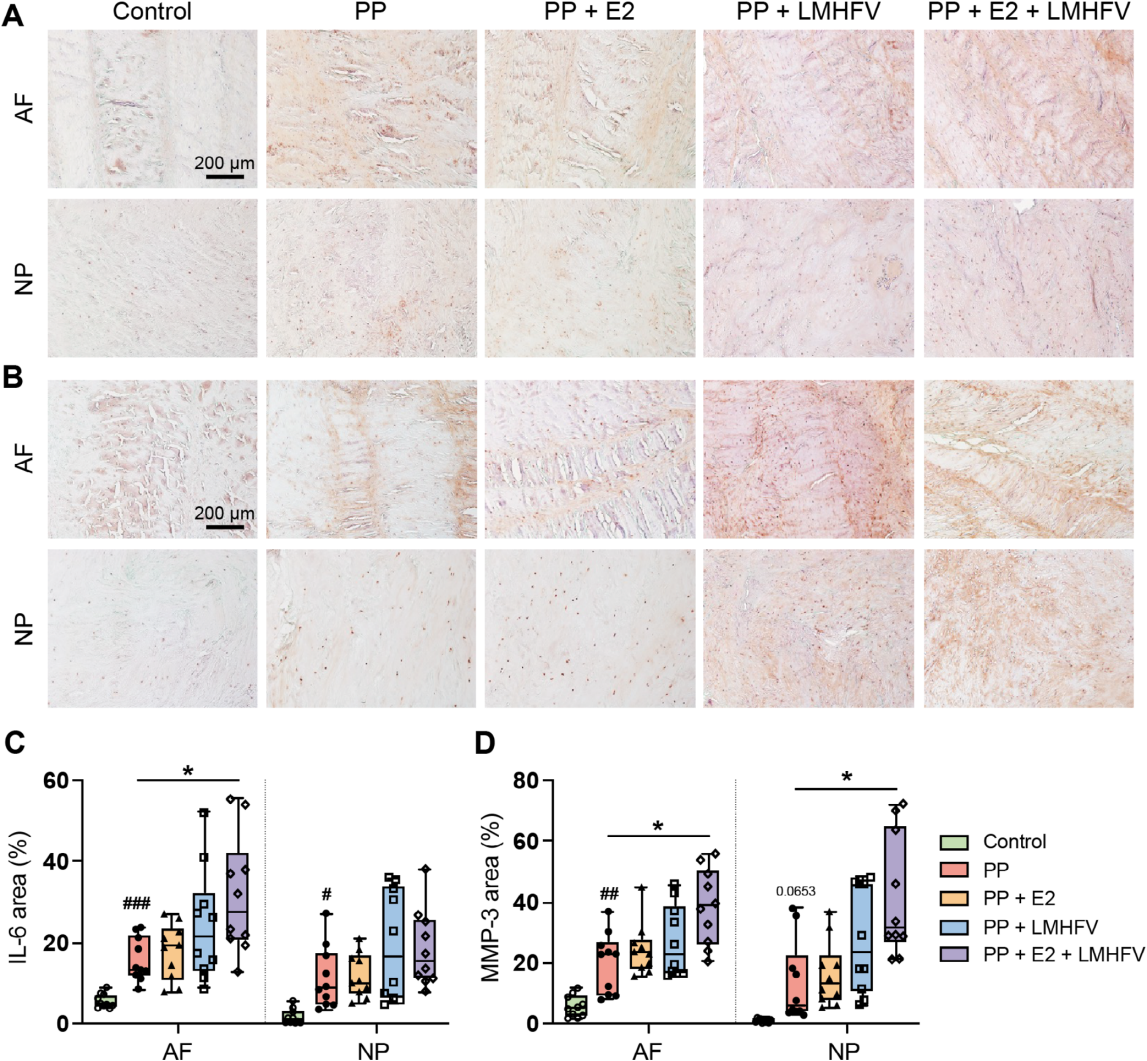
Representative images of (A) interleukin‐6 (IL‐6) and (B) matrix metalloproteinase 3 (MMP‐3) staining in the annulus fibrosus (AF) and nucleus pulposus (NP) of the control, papain‐degraded (PP), PP with estrogen (PP + E2), PP with low‐magnitude high‐frequency vibration (PP + LMHFV), and the combined PP + E2 + LMHFV groups on Day 21 of culture (scale bar, 200 μm). (C) IL‐6 and (D) MMP‐3 area (%). Data are presented as box plots showing the median ± interquartile range (*n* = 10 donors per group). ^#^ *p* < 0.05 (^#^Comparison to control); *p < 0.05 (*Comparison between treatment groups).

Immunohistochemical staining was used to assess protein levels of the inflammation‐associated cytokine IL‐6 and the matrix‐degrading enzyme MMP‐3 (Figure 6A,B). Quantification revealed significantly increased IL‐6 production in the PP‐treated group compared to controls in both AF and NP (*p* < 0.05; Figure 6C). Notably, the PP + E2 + LMHFV group showed even higher IL‐6 levels in the NP than PP alone (*p* < 0.05). Similarly, MMP‐3 expression in the AF was significantly elevated in all PP‐treated groups relative to controls (*p* < 0.05; Figure 6D), with a similar trend observed in the NP (*p* = 0.0653). In both AF and NP, MMP‐3 levels were further increased in the PP + E2 + LMHFV group compared to PP (*p* < 0.05).

### 3.6 AF Peel Stiffness and Strength

**FIGURE 7:**
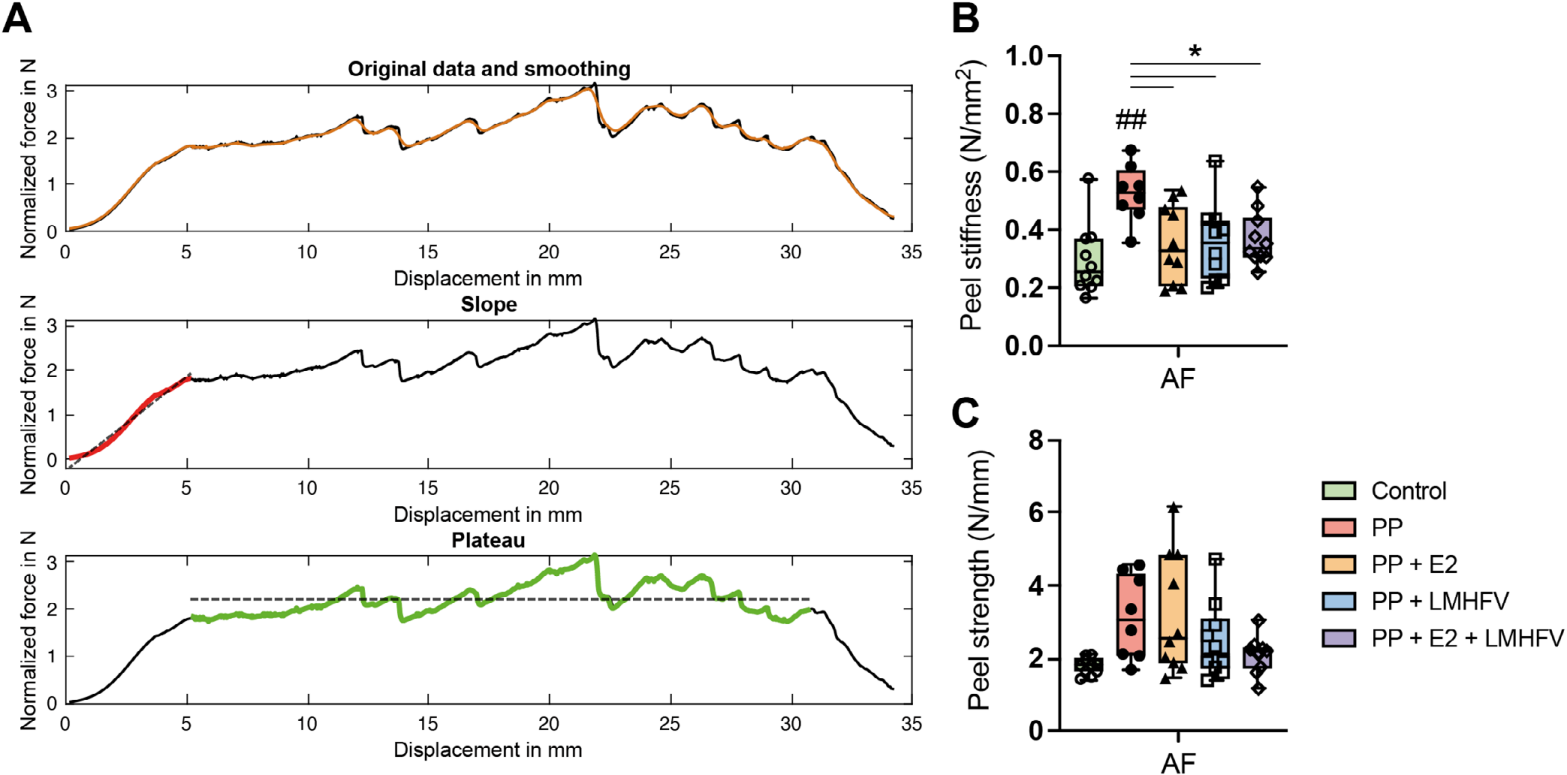
Mechanical characterization of the annulus fibrosus (AF) in disc samples across various treatment groups: Control, papain‐degraded (PP), PP with estrogen (PP + E2), PP with low‐magnitude high‐frequency vibration (PP + LMHFV), and the combination of PP + E2 + LMHFV. (A) Exemplary force/displacement curve (N/mm), with the slope highlighted in *red* and the plateau region in green. (B) Peel stiffness determined from the slope of the force/displacement curve. (C) Peel strength calculated using the plateau of the force/displacement curve. Data are presented as *xy* plots or box plots (*n* = 8–10 donors per group). ^#^ *p* < 0.05 (^#^Comparison to control); *p < 0.05 (*Comparison between treatment groups).

An exemplary force/displacement curve (N/mm) is depicted in Figure 7A. Peel stiffness was determined from the slope of the force/displacement curve and peel strength was calculated as the average of the plateau. Compared to the control, PP treatment alone led to a significant increase of approximately 78% in peel stiffness (*p* < 0.01), as shown in Figure 7B. However, additional treatment with E2, LMHFV, or their combination significantly attenuated this increase (*p* < 0.05), reversing the PP‐induced effect. In contrast, no significant differences in peel strength were observed among the treatment groups (Figure 7C).

## 4 Discussion

Our study showed that, in an advanced PP‐induced degenerative ex vivo IVD model, application of LMHFV (45 Hz, 0.3 g, 20 min/day, 5 days/week for about 2 weeks) compromises cell viability, amplifies inflammatory and catabolic activity, and accelerates matrix loss. In contrast, E2 treatment was insufficient to counteract the effects of PP and LMHFV.

PP digestion effectively induced degeneration, characterized by GAG depletion, increased IL‐6 and MMP‐3 expression, and biomechanical alterations indicative of matrix degradation and inflammation, consistent with previous enzyme‐based degeneration models [31, 36, 45]. The observed increase in AF peel stiffness is most likely due to proteoglycan loss associated with accumulation of collagen type I and fibrosis [47, 48]. Overall, PP provides a reliable and reproducible method for generating a degeneration‐mimicking IVD model suitable for therapeutic evaluation [31, 36].

E2 is often regarded as protective against IVDD, with reports showing beneficial effects on matrix metabolism and cell survival under healthy or mildly degenerative conditions [15, 24, 29, 32, 33, 34]. For the organ culture model, IVDs from male cattle were used. Although E2 is a sex‐specific factor, the experiments were performed ex vivo, thereby minimizing systemic hormonal influences, including endogenous estrogen levels. Moreover, estrogen receptors (ERα and ERβ) have been identified in both annulus fibrosus and NP cells from male and female human samples [11, 16, 17, 29], suggesting that IVD cells from both sexes can respond to estrogen stimulation. However, in our severely degenerated model, E2 failed to counteract matrix loss or inflammatory activation. This may reflect reduced estrogen receptor expression and signaling following PP digestion, as previously observed in degenerated IVDs [11, 16]. E2's protective effects are mediated, for instance, via ER‐dependent PI3K/Akt and ERK/MAPK pathways [49, 50], and their downregulation likely limits responsiveness. Additionally, E2 bioavailability in 3D organ cultures may be lower than in monolayer or in vivo models, reducing its efficacy [51].

LMHFV is increasingly used therapeutically for bone and muscle health, particularly in postmenopausal females [19, 52]. In healthy IVD models, LMHFV has been shown to transiently induce anabolic gene expression [24] and preserve disc height [25]. In the present study, we confirmed that LMHFV effectively reached the center of the IVD. In PP‐digested discs, LMHFV increased the expression of catabolic genes in both the AF and NP, consistent with reports that prolonged LMHFV can promote joint degeneration [27, 32, 33]. Interestingly, LMHFV also increased DNA content, reflecting enhanced cell proliferation as previously reported in mesenchymal stem cells and osteoblasts [53], but concurrently reduced cell viability in the NP, indicating a divergence between proliferation and survival, possibly due to metabolic exhaustion in a nutrient‐limited environment [54]. Additionally, LMHFV stimulated *ACAN* expression and GAG production, consistent with previous in vivo findings of short‐term LMHFV exposure in mice [24]. The increased cellularity observed in the LMHFV groups may partly reflect enhanced nutrient transport rather than a purely mechanobiological effect. The IVD is an avascular tissue in which nutrient supply and waste removal rely primarily on diffusion but can be significantly influenced by mechanical loading–induced fluid flow and convection [54]. Even short periods of dynamic loading may transiently enhance solute transport and metabolic exchange within the IVD [55, 56], potentially contributing to increased cell proliferation and DNA content. However, such transient improvements in transport are likely insufficient to sustain long‐term cell viability in the NP.

From a mechanobiological perspective, NP cells are well adapted to hydrostatic pressure, whereas distortional shear stresses promote catabolic and inflammatory responses [57]. In the present degeneration model, the loss of physiological intradiscal pressure following PP treatment likely shifts the mechanical environment toward *unfavorable* loading conditions, potentially limiting the effectiveness of interventions such as LMHFV and E2. Restoration of the intradiscal pressure by an injectable biomaterial has been shown to promote anabolic and regenerative responses and to improve NP cell vitality [58]. This suggests that re‐establishing IVD pressurization may be a critical factor for achieving successful therapeutic outcomes.

Interestingly, combined E2 and LMHFV treatment increased DNA content, similar to LMHFV alone, while maintaining NP cell viability, as observed with E2. This may suggest partial activation of E2's antiapoptotic pathways, possibly via PI3K/Akt/mTOR signaling [59]. Nonetheless, matrix catabolism was further elevated than in PP digestion alone, and no improvement in matrix composition was observed, indicating that combined treatment was insufficient to overcome the effects of PP digestion. These findings contrast with previous observations in mildly degenerated IVDs, where combined E2 + LMHFV treatment had a protective effect against IVDD, reducing cell loss and suppressing IL‐6 production relative to LMHFV alone [29]. This disparity highlights the potential importance of degeneration stage in determining the cellular response to mechanical and hormonal interventions. Moreover, this reinforces the hypothesis that E2 is more effective in early or mild stages of degeneration, rather than in extensively damaged tissue.

Mechanotransduction in IVD cells is a complex process involving integrin‐mediated signaling, cytoskeletal remodeling, and calcium influx, all of which influence downstream gene expression and extracellular matrix remodeling [60, 61]. Importantly, the biological effects of vibration on the IVD are highly dependent on specific parameters such as frequency, amplitude, and duration, which collectively influence whether the mechanical stimulus produces beneficial anabolic effects or detrimental catabolic responses [24, 32, 62]. These factors may determine whether the mechanical input elicits anabolic or catabolic responses, underscoring the need for tailored protocols based on the stage of IVDD and tissue integrity. Taking this into account, our study includes limitations including the use of PP to enzymatically induce IVD degeneration, reflecting a chemically induced, acute injury rather than the gradual, multifactorial degeneration observed in humans (e.g., aging, endplate changes, nutrient deficiency) [63]. Second, the ex vivo culture lacks systemic influences such as vascular supply, immune cell influx, hormonal fluctuations, and long‐term remodeling capacity, which limits translation to in vivo conditions [64]. Lastly, a single vibration protocol (45 Hz, 0.3 g, 20 min/day for 2 weeks) and single E2 dose were applied. Different frequencies, amplitudes, durations, or hormone concentrations might yield distinct outcomes, limiting the direct applicability of results to human post‐menopausal IVDs. Despite these caveats, the study provides valuable mechanistic insights into how hormonal and mechanical interventions interact under advanced degenerative conditions.

## 5 Conclusions and Future Perspectives

PP degradation effectively simulated advanced IVDD, leading to substantial loss of physiological disc pressurization and GAG content, increased inflammation, and structural remodeling. The increase in cellularity with LMHFV may partly reflect transient improvements in nutrient transport; however, E2 and LMHFV were insufficient to reverse degenerative changes, and in combination exacerbated catabolic responses in specific regions. Taking this into consideration, our findings suggest that the use of LMHFV as an approach against osteoporosis should be approached with caution. Future research should investigate combination therapies involving hormonal, mechanical, metabolic, and antiinflammatory agents, ideally using models that more accurately reflect the progressive nature of human disc degeneration.

## Author Contributions

**Hans‐Joachim Wilke**, **Anita Ignatius**, **Melanie Haffner‐Luntzer**, **Cornelia Neidlinger‐Wilke**, and **Graciosa Quelhas Teixeira:** conceptualization. **Jan Ulrich Jansen**, **Felizitas Figel**, **Franziska Widmayer**, **Morten Vogt**, and **Maria Ahrens:** data curation, formal analysis, methodology, writing – original draft. **Hans‐Joachim Wilke**, **Anita Ignatius**, **Melanie Haffner‐Luntzer**, and **Cornelia Neidlinger‐Wilke:** writing – review and editing. **Graciosa Quelhas Teixeira:** project administration, supervision, funding acquisition, writing – original draft, review and editing. All authors have read and agreed to the published version of the manuscript.

## Funding

This work was supported by Universität Ulm (KNMÜ.031.02, Hertha‐Nathorff‐Programme).

## Ethics Statement

The authors have nothing to report.

## Conflicts of Interest

The authors declare no conflicts of interest.

## Supporting information


**Figure S1:** Cell viability across different treatment groups: control, papain‐treated (PP), PP with estrogen (PP + E2), PP with low‐magnitude high‐frequency vibration (PP + LMHFV), and the combination of PP + E2 + LMHFV. Representative confocal microscopy images of live/dead staining in the (A) annulus fibrosus (AF) and (B) nucleus pulposus (NP) after 21 days of organ culture, showing cell viability. Dead cells are stained red, while live cells appear green (scale bars, 200 μm).

## Data Availability

The data that support the findings of this study are available from the corresponding author upon reasonable request.
