# Supplementary material for: Detrimental Effect of Low‐Magnitude High‐Frequency Vibration in an Ex Vivo Model of Intervertebral Disc Degeneration Despite Estrogen Treatment
Source: JOR Spine. 2026 Apr 22;9(2):e70183. doi: 10.1002/jsp2.70183 (PMC13101075; doi:10.1002/jsp2.70183)
Supplement: Supplementary file 1 — Figure S1: Cell viability across different treatment groups: control, papain‐treated (PP), PP with estrogen (PP + E2), PP with low‐magnitude high‐frequency vibration (PP + LMHFV), and the combination of PP + E2 + LMHFV. Representative confocal microscopy images of live/dead staining in the (A) annulus fibrosus (AF) and (B) nucleus pulposus (NP) after 21 days of organ culture, showing cell viability. Dead cells are stained red, while live cells appear green (scale bars, 200 μm). [file JSP2-9-e70183-s001.docx]

**Supplementary Data**

**
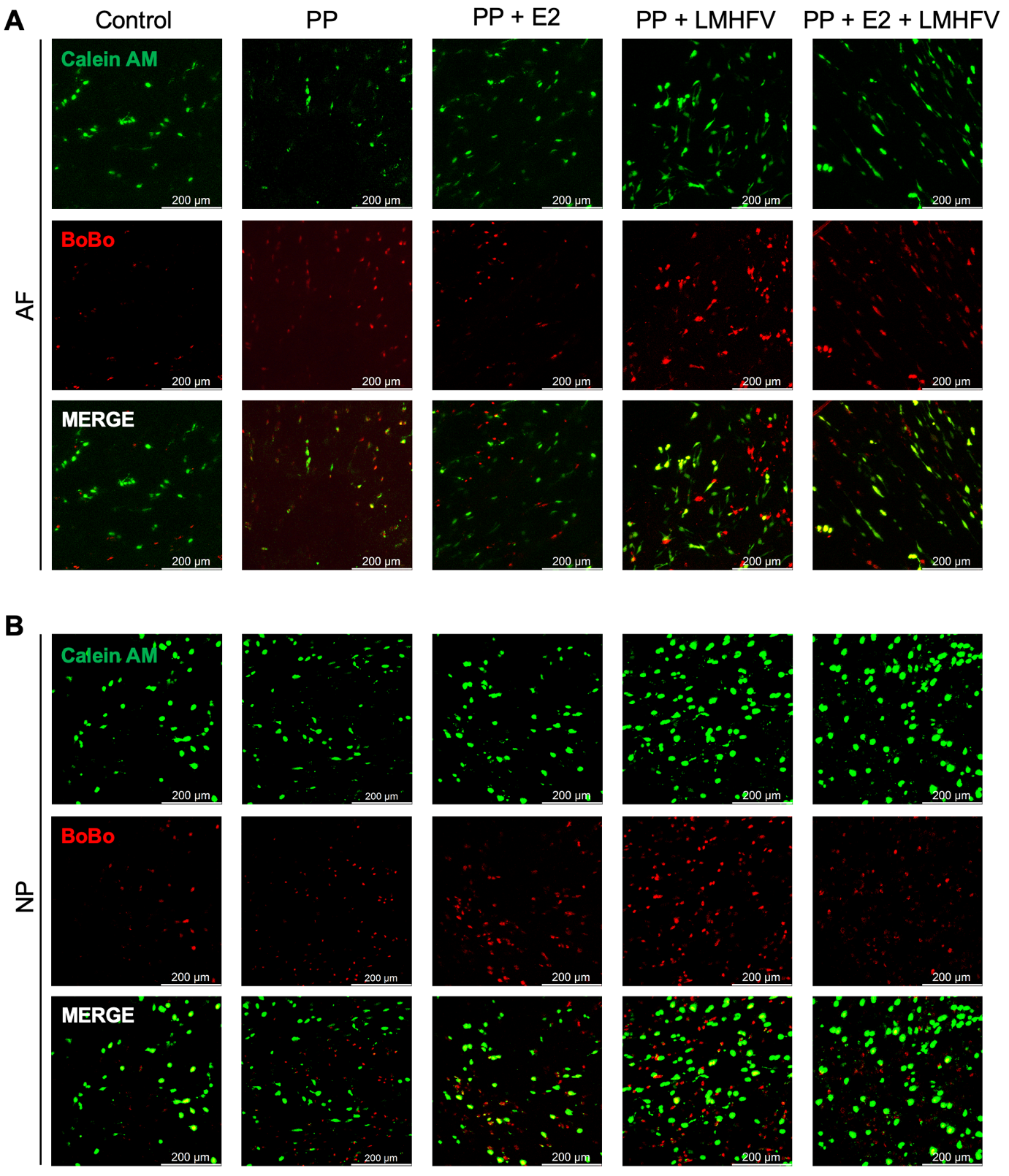
**

**Figure S1.** Cell viability across different treatment groups: control, papain-treated (PP), PP with oestrogen (PP + E2), PP with low-magnitude high-frequency vibration (PP + LMHFV), and the combination of PP + E2 + LMHFV. Representative confocal microscopy images of live/dead staining in the (A) annulus fibrosus (AF) and (B) nucleus pulposus (NP) after 21 days of organ culture, showing cell viability. Dead cells are stained red, while live cells appear green (scale bars, 200 μm).
